# Introduction of the *Anopheles bancroftii* Mosquito, a Malaria Vector, into New Caledonia

**DOI:** 10.3201/eid2403.171689

**Published:** 2018-03

**Authors:** Morgane Pol, Sosiasi Kilama, Sandy Duperier, Marie-Estelle Soupé-Gilbert, Elodie Calvez, Nicolas Pocquet

**Affiliations:** Institut Pasteur, Noumea, New Caledonia (M. Pol, S. Kilama, M.-E. Soupé-Gilbert, E. Calvez, N. Pocquet);; Directorate of Health and Social Affairs, Noumea (S. Duperier)

**Keywords:** Anopheles bancroftii, malaria vector, mosquito introduction, New Caledonia, Pacific region, vector-borne infections, mosquitoborne infections, parasites, malaria

## Abstract

In June 2017, an *Anopheles* mosquito species was detected in New Caledonia. Morphologic identification and genomic sequencing revealed that the specimens tested belong to *An. bancroftii* genotype A1. This introduction underscores the risk for local malaria transmission and the vulnerability of New Caledonia to vector introduction.

New Caledonia, a French island in the southern Pacific Ocean, had been free of *Anopheles* mosquito species (*1*). The absence of all potential vectors of human *Plasmodium* spp. made New Caledonia free of malaria transmission. However, this situation is fragile because many neighboring countries (e.g., Papua New Guinea, Solomon Islands, and Vanuatu) are endemic for malaria and have highly competent malaria vectors (*2*) that could be introduced into New Caledonia. Entomologic surveillance conducted since 1979 has never found any *Anophelinae* mosquitoes in New Caledonia (*3*). In June 2017, however, a survey indicated the introduction of *An. bancroftii* mosquitoes into New Caledonia. This discovery prompted us to strengthen entomologic surveillance in the detection area to evaluate the situation. 

During June 7–September 8, 2017, we trapped and identified 3,181 mosquitoes, including 27 *An. bancroftii* mosquitoes. All *An. bancroftii* mosquitoes were trapped in an area of 6 km^2^, out of 50 km^2^ covered by the survey. The first *An. bancroftii* specimen was trapped by using a Biogents Sentinel Trap (Biogents AG, Regensburg, Germany) at a plant nursery. Another specimen was detected 3 km away at the international airport, where traps designed at the US Centers for Disease Control and Prevention (CDC), equipped with UV light and CO_2_, had been set. All other specimens were collected at the plant nursery by using CDC traps (14 females and 3 males) or the human landing catches technique (8 females). The collector (S.K.) noticed that, when he was in the shade, the *An. bancroftii* female mosquitoes were aggressive even during daytime (e.g., 2:00–4:00 pm). Despite all investigation efforts, no *Anopheles* mosquito larvae were found.

We used 2 morphologic identification keys to determine species (*4*,*5*). Both keys identified the individual specimens captured as belonging to the species *An. bancroftii*. The 2 species of the *bancroftii* group (*An. bancroftii* and *An. pseudobarbirostris*) can only be distinguished by pale patches on the wing fringe of the adult females. Because this morphologic criterion is controversial (*6*), we used 2 collected specimens to improve identification through sequencing. The primers we used distinguish between different genotypes in the *An. bancroftii* group by means of an internal transcribed spacer 2 gene sequence analysis (*7*), which indicated that both specimens collected in the nursery plant (GenBank accession nos. MF716525 and MF716526) match at 100% with *An. bancroftii* genotype A1 (GenBank accession no. AF203381; Figure). A 2001 study showed that *An. bancroftii* genotype A1 mosquitoes were found only in the Northern Territory of Australia, whereas other genotypes were found in Queensland, Australia, or in Papua New Guinea (Technical Appendix Figure) (*7*). This genetic analysis provides a small clue regarding the origin of the introduction of this species but does not indicate its route. This genotype might have been introduced in other countries during 2001–2017 before reaching New Caledonia.

**Figure:**
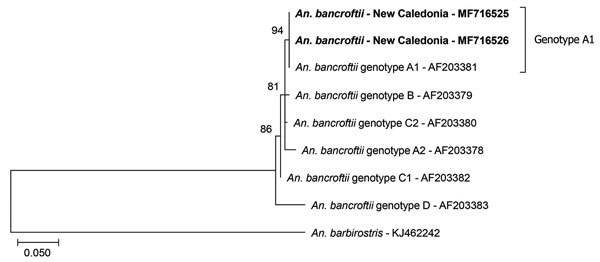
Phylogenetic analysis of *Anopheles* mosquito species introduced in New Caledonia. Phylogenetic trees were generated by the maximum-likelihood method based on the Kimura 2-parameter model using MEGA7 (http://www.megasoftware.net). Percentage bootstrap values shown at the nodes were calculated with 1,000 replicates. Bold indicates strains isolated in this study. Scale bar indicates nucleotide substitutions per site.

In Papua New Guinea, *An. bancroftii* mosquitoes are considered a secondary vector of malaria because of their weak anthropophilic feeding behavior and their small numbers in the regions studied. Furthermore, when investigated, the proportion of sporozoite-positive specimens was low (<1% in ELISA targeting circumsporozoite protein) (*8*,*9*). In the Northern Territory of Australia, where only genotype A1 is present (*7*), *An. bancroftii* mosquitoes are described as a major pest species because females readily and aggressively bite humans (*6**,**7*). This observation concurs with our preliminary observations made in New Caledonia, which might result in higher numbers of human–vector contacts than reported in Papua New Guinea. Considering that an average of 4 imported malaria cases were reported annually during 2000–2015 in New Caledonia (*10*), the introduction of a potential malaria vector in a new environment raises the specter of long-term local malaria transmission risk. In case of establishment, the risk for local transmission will be assessed by investigating the vector competence of the introduced genotype.

Although the bioecology of *An. bancroftii* mosquitoes remains poorly known in New Caledonia, the larval habitats have been previously described as floodplains and freshwater swamps (*6*). These ecosystems are abundant in the proximity of the plant nursery were adult mosquito specimens where initially detected. Although no larvae have been found, 27 adults were trapped during a period of 4 months, suggesting efficient colonization. Establishment of the species is a main concern, and sustainable entomologic surveillance might assist in the design and evaluation of an eradication plan.

In summary, we describe the introduction of *An. bancroftii* genotype A1 mosquitoes into New Caledonia, a territory previously known as free from *Anopheles* mosquito species. Although this species is not the most competent malaria vector, this sentinel event points to New Caledonia’s vulnerability to the introduction of more competent vectors. Furthermore, because of its localization, its economic status, and the sea and air connections it shares with other Pacific islands, New Caledonia functions as a hub in the region. If *An. bancroftii* mosquitoes settle in New Caledonia, they could further spread to other *Anopheles*-free territories in the South Pacific.

Technical Appendix Known distribution of *Anopheles bancroftii* mosquitos and localization of genotypes.
