# Supplementary material for: Introduction of the Anopheles bancroftii Mosquito, a Malaria Vector, into New Caledonia
Source: Emerg Infect Dis. 2018 Mar;24(3):604–5. doi: 10.3201/eid2403.171689 (PMC5823349; doi:10.3201/eid2403.171689)
Supplement: Technical Appendix — Known distribution of Anopheles bancroftii mosquitos and localization of genotypes. [file 17-1689-Techapp-s1.pdf]

Article DOI: https://doi.org/10.3201/eid2403.171689

Introduction of the *Anopheles bancroftii* Mosquito, a Malaria Vector, into New Caledonia

Technical Appendix

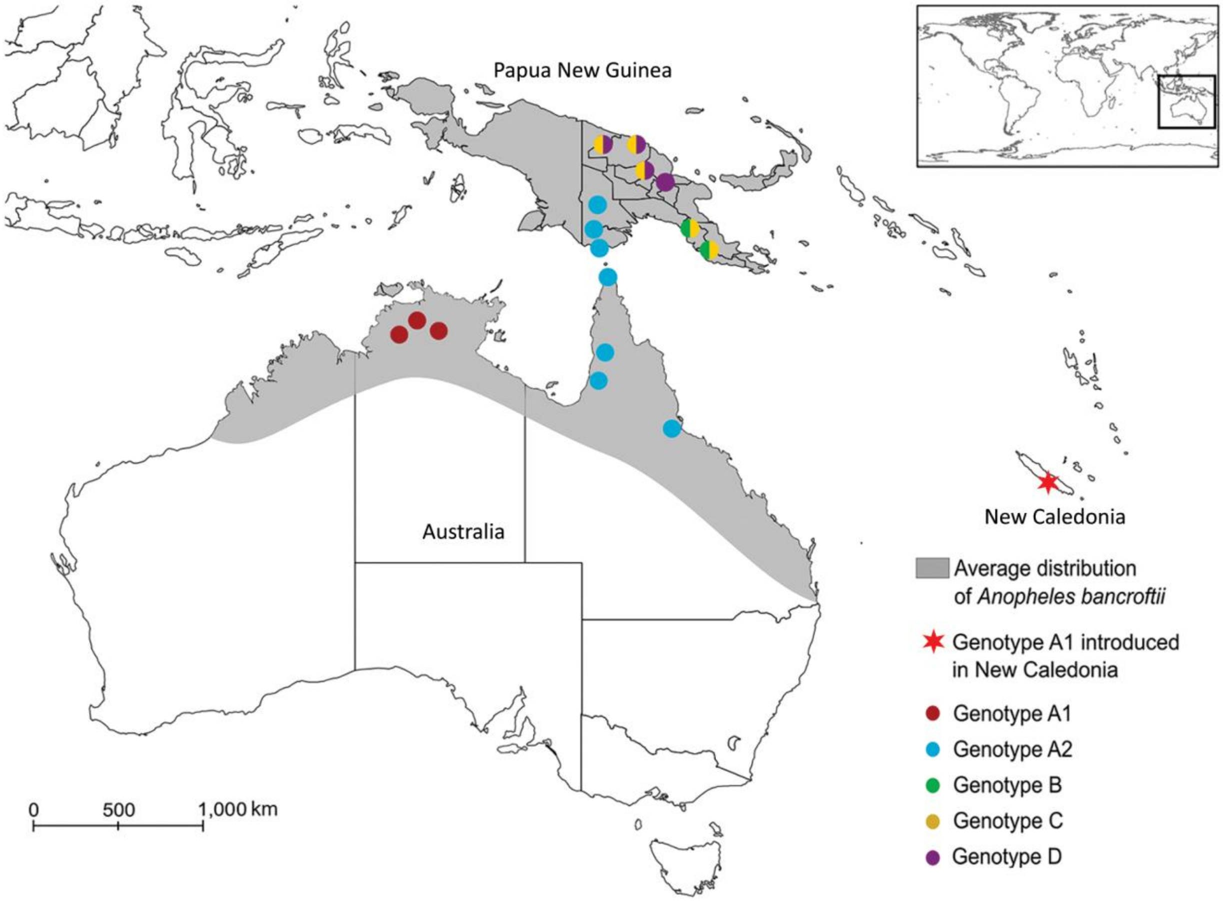

Technical Appendix Figure. Known distribution of *Anopheles bancroftii* mosquitos and localization of genotypes. Gray shading indicates approximate known distribution of *An. bancroftii* mosquitos, according to previous publications (*1*–*3*). Star indicates the localization of the genotype A1 introduced in 2017 in New Caledonia, as described in this study. Circles indicate locations of the different genotypes studied in 2001 (*3*). Inset shows location of primary map in the southern Pacific Ocean.

References

1. Lee D, Hicks M, Griffiths M, Debenham M, Bryan J, Russell R, et al. In: Debenham ML, editor. The *Culicidae* of the Australasian Region. Volume 5. Canberra (Australia): Australian Government Publishing Service; 1987.

2. Beebe NW, Russell TL, Burkot TR, Lobo NF, Cooper RD. The systematics and bionomics of malaria vectors in the southwest Pacific. In: Manguin S, editor. *Anopheles* mosquitoes–new insights into malaria vectors. Rijeka (Croatia): InTech; 2013. p. 357–94.

3. Beebe NW, Maung J, van den Hurk AF, Ellis JT, Cooper RD. Ribosomal DNA spacer genotypes of the *Anopheles bancroftii* group (Diptera: Culicidae) from Australia and Papua New Guinea. Insect Mol Biol. 2001;10:407–13. PubMed http://dx.doi.org/10.1046/j.0962-1075.2001.00278.x
